# Sphingolipid-Containing Outer Membrane Vesicles Serve as a Delivery Vehicle To Limit Macrophage Immune Response to Porphyromonas gingivalis

**DOI:** 10.1128/IAI.00614-20

**Published:** 2021-03-17

**Authors:** Fernanda G. Rocha, Gregory Ottenberg, Zavier G. Eure, Mary E. Davey, Frank C. Gibson

**Affiliations:** a Department of Oral Biology, College of Dentistry, University of Florida, Gainesville, Florida, USA; Georgia Institute of Technology School of Biological Sciences

**Keywords:** *Porphyromonas gingivalis*, host-pathogen interactions, outer membrane vesicles, periodontitis, sphingolipids

## Abstract

Sphingolipids (SLs) are essential structural components of mammalian cell membranes. Our group recently determined that the oral anaerobe Porphyromonas gingivalis delivers its SLs to host cells and that the ability of P. gingivalis to synthesize SLs limits the elicited host inflammatory response during cellular infection. As P. gingivalis robustly produces outer membrane vesicles (OMVs), we hypothesized that OMVs serve as a delivery vehicle for SLs, that the SL status of the OMVs may impact cargo loading to OMVs, and that SL-containing OMVs limit elicited host inflammation similar to that observed by direct bacterial challenge. Transwell cell culture experiments determined that in comparison to the parent strain W83, the SL-null mutant elicited a hyperinflammatory immune response from THP-1 macrophage-like cells with elevated tumor necrosis factor alpha (TNF-α), interleukin 1β (IL-1β), and IL-6. Targeted assessment of Toll-like receptors (TLRs) identified elevated expression of TLR2, unchanged TLR4, and elevated expression of the adaptor molecules MyD88 and TRIF (Toll/IL-1 receptor domain-containing adaptor-inducing beta interferon) by SL-null P. gingivalis. No significant differences in gingipain activity were observed in our infection models, and both strains produced OMVs of similar sizes. Using comparative two-dimensional gel electrophoresis, we identified differences in the protein cargo of the OMVs between parent and SL-null strain. Importantly, use of purified OMVs recapitulated the cellular inflammatory response observed in the transwell system with whole bacteria. These findings provide new insights into the role of SLs in P. gingivalis OMV cargo assembly and expand our understanding of SL-OMVs as bacterial structures that modulate the host inflammatory response.

## Abstract (precis)

Sphingolipids (SLs) are essential structural components of mammalian cell membranes. Our group recently determined that the oral anaerobe Porphyromonas gingivalis delivers its SLs to host cells and that the ability of P. gingivalis to synthesize SLs limits the elicited host inflammatory response during cellular infection.

## INTRODUCTION

Microbial outer membrane vesicles (OMVs), also referred to extracellular vesicles, are spherical nanosized proteoliposomes that are generated by vesiculation of the bacterial outer membrane. OMVs have been shown to represent a key mode of interkingdom communication between bacteria and host tissues (1). OMVs range in size from 20 to 400 nm, and they are comprised of a lipid bilayer containing lipopolysaccharide, outer membrane proteins, and other bioactive effector molecules such as periplasmic and cytosolic proteins, nucleic acids, and immunomodulatory factors (2, 3). The mechanism of OMV production is partially understood (1). It is also known that the biogenesis of OMVs is beneficial for bacterial pathogens, as these structures are known to deliver multiple virulence factors to the host. In addition, OMVs concentrate and protect virulence determinants from host degradative process such as proteases, confer antibiotic resistance, and facilitate biofilm formation (2, 4). Several studies have demonstrated that purified OMVs can enhance microbial pathogenicity by triggering the release of proinflammatory cytokines, as well as promoting neutrophil migration and disruption of epithelial cell junctions (5, 6). Not surprisingly, some beneficial effects have been linked to OMV production by bacteria from the host gut microbiota (4).

Periodontitis is among the most common bacterium-elicited chronic inflammatory diseases of humans (7). In susceptible individuals, bacteria in the subgingival biofilm promote dysregulated inflammation and microbial dysbiosis that results in the progressive destruction of soft and hard tissues that support the teeth, which is irreversible (8). Locally, one of the characteristics of sites affected by periodontitis is the increase of Gram-negative anaerobes, such as Porphyromonas gingivalis, Tannerella forsythia, and Treponema denticola, that are abundant in specific virulence factors, including gingipains, lipopolysaccharide, capsular polysaccharide, lipoproteins, and other molecules (9, 10). Interestingly, several bacteria associated with periodontal disease, including P. gingivalis, are known OMV producers (6, 11, 12).

There is significant interest as to how P. gingivalis evolves and contributes to the changing inflammatory milieu from periodontal health to disease. Indeed, although this organism is closely associated with periodontal disease, this bacterium is found in subgingival plaque during periodontal health (13), suggesting that the host does not always responds to P. gingivalis as a pathogen. An area of intense focus has been the ability of P. gingivalis to perform targeted immunosuppression and immune evasion by deploying molecules, including its gingipains, atypical lipopolysaccharide (LPS), and other factors to disrupt the host immune response (14–16). Recently, our group reported that P. gingivalis possesses an additional layer of immunosuppressive ability that is mediated by its ability to synthesize sphingolipids (SLs). Furthermore, and intriguingly, the SLs are delivered to host cells in a contact-independent manner (17). Considering that P. gingivalis is known to be a robust OMV producer, here we hypothesized that SL-containing OMVs (SL-OMVs) may serve as a unique delivery platform and that the SL-OMVs can limit the host immune response.

In this study, we purified OMVs from SL-containing or SL-null P. gingivalis and assessed the protein content of these vesicles. In addition, using a macrophage model cell culture system, we characterized the immunomodulatory effect of whether P. gingivalis synthesizes SLs on the host immune response. Moreover, we demonstrate using THP-1 cells that in pure form SL-OMVs and OMVs lacking SLs recapitulate the host immune response observed in response to parent and mutant P. gingivalis infection.

## RESULTS

### SL synthesis limits the inflammatory response of immune cells to P. gingivalis in a contact-independent manner.

**FIG 1:**
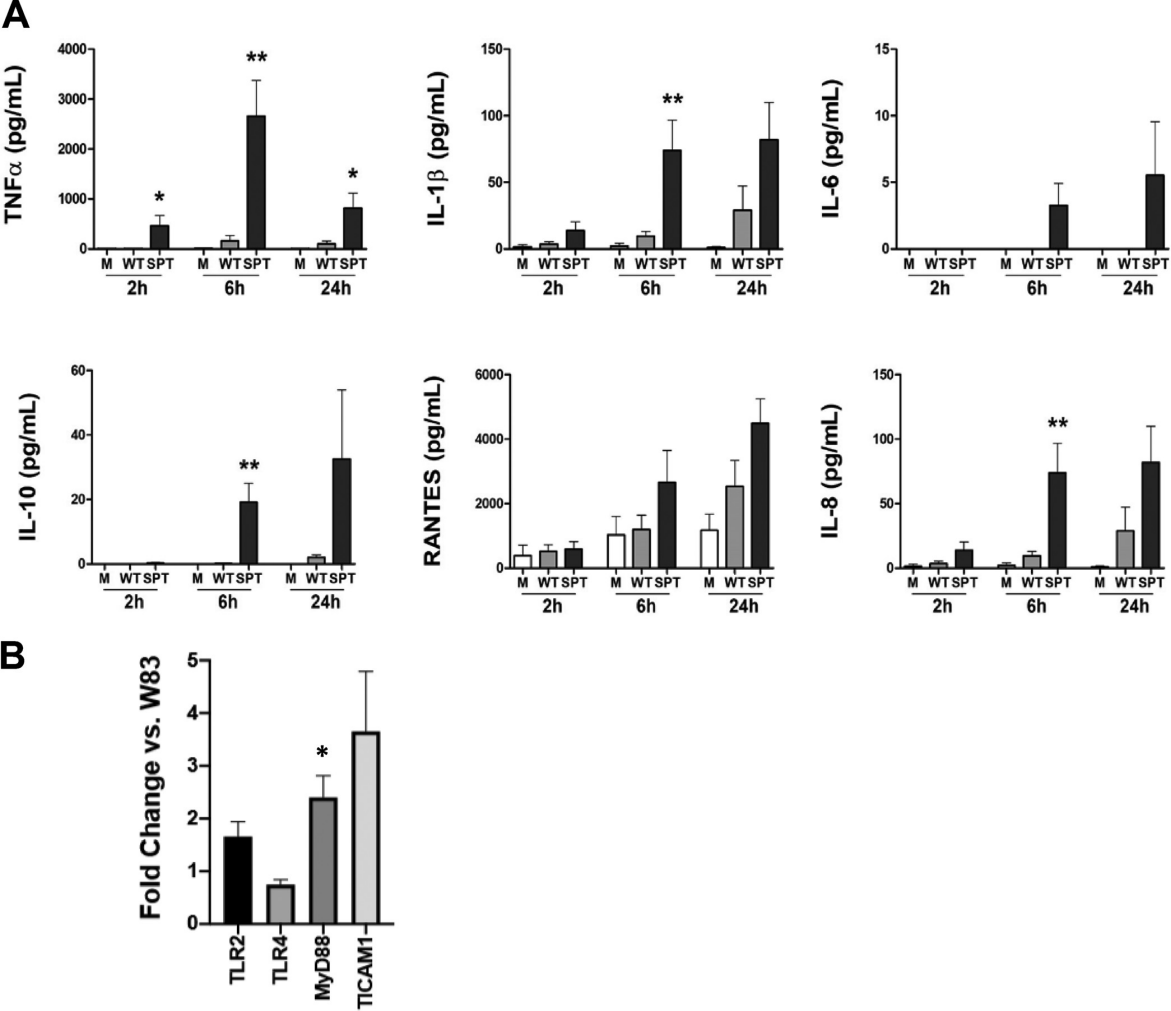
P. gingivalis sphingolipids (SLs) suppress host immune response independently of direct contact. Supernatant fluids were collected from PMA-activated human THP-1 cells placed in the lower well of a transwell system and to the upper well was added either P. gingivalis W83 (WT; gray bars) or the P. gingivalis W83 SL-null mutant (SPT-; black bars) at 2, 6, and 24 h of culture, and the levels of TNF-α, IL-1β, IL-6, IL-10, RANTES, and IL-8 were measured by multiplex immunoassay (A). Medium alone (M; white bars) served as unchallenged control. (B) Transcriptional levels of TLR pathway genes from THP-1 cells cultured in transwells, with medium, P. gingivalis W83 WT, or P. gingivalis SPT- for 2 h. Expression data were normalized to β-actin, and fold change in TRL pathway genes are presented for SPT relative to strain W83. Data are presented as means plus standard errors of the means (SEM) (error bars) (*n* = 3 independent experiments). Values that are significantly different from the value for WT P. gingivalis using unpaired *t* test are indicated by asterisks as follows: *, *P* < 0.05; **, *P* < 0.01.

To investigate the immunoregulatory effect of P. gingivalis SL synthesis in cell culture without direct contact with host cells, we utilized a 0.4-μm-pore transwell system; hence, the OMVs (<400 nm) can cross the transwell membrane, while preventing direct bacterium-host cell contact. We observed that stimulation with the SL-null mutant (serine palmitoyl transferase null mutant [SPT- mutant]) elicited a more robust immune response from THP-1 cells compared to the response elicited by the wild type (WT) (Fig. 1A). As early as 2 h after initiation of transwell coculture, significantly higher levels of TNF-α were measured in supernatant fluids from THP-1 cells cultured with SL-null P. gingivalis compared to the parent (*P* < 0.05; Fig. 1A), a trend that was maintained through the 24-h infection period. There was also a significant increase in the levels of IL-1β and IL-8 by 6 h elicited by the SL-null mutant (*P* < 0.05 for all by *t* test). The trend of hyperinflammatory response when SLs were not synthesized could also be observed in the measurements of IL-6, IL-10, and RANTES (*r*egulated upon *a*ctivation, *n*ormal *T* cell *e*xpressed and *s*ecreted) (Fig. 1A). These data support the concept that SL production limits the inflammatory response of immune cells to P. gingivalis and that this immunoregulatory capacity does not require bacterium-to-host cell contact.

Considering the differences in the cytokines and chemokine levels in the THP-1 cell supernatants, understanding that some P. gingivalis SLs are sensed by Toll-like receptors (TLRs) (18), and the knowledge that OMV activation of host cells is mediated in part by TLRs (19), we subsequently evaluated gene expression of selected TLR pathway members: TLR2, TLR4, MyD88, and TICAM (Toll-interleukin 1 receptor-containing adaptor molecule 2)/TRIF (Toll/IL-1 receptor domain-containing adaptor-inducing beta interferon). In comparison to WT P. gingivalis, stimulation with the SPT- mutant resulted in increase of TLR2 gene expression (*P* < 0.05) without notable change in the expression of TLR4 (Fig. 1B). Investigating the expression of the key adaptor molecules responsible for TLR signaling, we observed increased expression of MyD88 and TICAM1/TRIF when THP-1 cells were stimulated with the SPT mutant, in comparison to WT (*P* < 0.05 and *P* < 0.05, respectively; Fig. 1B). Although further investigation is needed to confirm the participation of TLR2, TLR4, MyD88, and TICAM/TRIF signaling pathways, these data support that when P. gingivalis can synthesize SLs, this limits the ability of the host cell to mount a robust immune response to this organism. Moreover, since the regulation occurred in a transwell system, the most likely delivery mechanism is via SL-containing OMVs.

### NanoSight characterization of P. gingivalis OMVs from SL-producing (WT) and SL-null mutant (SPT) and protein profiles.

**FIG 2:**
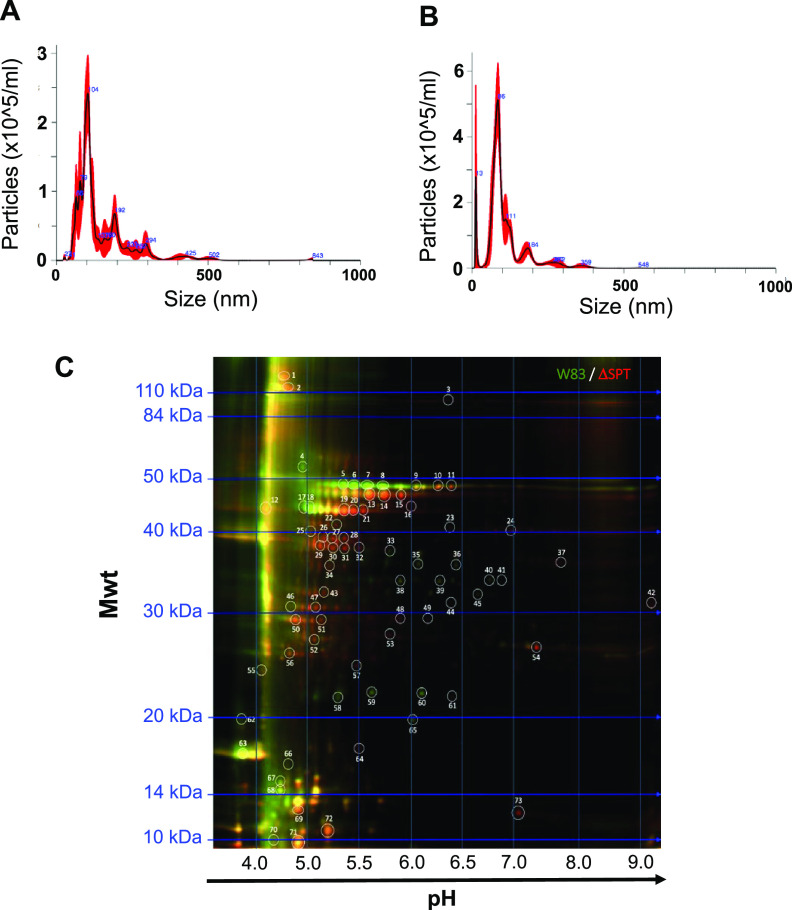
Characterization of P. gingivalis OMVs. NanoSight analysis of OMVs from WT P. gingivalis W83 (A) and P. gingivalis ΔSPT mutant (B). (C) Comparative fluorescent two-dimensional gel of OMV protein cargo from WT P. gingivalis W83 (green spots) and the ΔSPT mutant (red spots). Proteins common in OMVs of the WT and ΔSPT strains are yellow. Spots 5, 6, 7, 8, 50, 60 and 65 were selected for further analysis (see Table 1). Mwt, molecular weight.

OMVs were isolated from the culture medium of the WT and SPT- mutant and analyzed using NanoSight, and the size distribution was comparable between WT and the SPT- mutant (Fig. 2A). Further, the SPT- mutant was found to be more proficient than the WT in OMV production (Fig. 2B), suggesting that the inability to synthesize SLs may lead P. gingivalis to increase the production of other types of OMVs.

To begin to understand how SL synthesis may influence P. gingivalis OMV cargo, comparative two-dimensional gel electrophoresis was used to evaluate the proteins present in purified OMVs isolated from the WT and the SPT- mutant. Remarkably, several protein spots (spots 5, 6, 7, 8, 60, and 65) that were at higher abundance in the SL-containing OMVs in comparison with the SL-null OMVs (Fig. 2C; Table 1), were all identified as PG1881. PG1881 is a predicted pilin lipoprotein recently reported to be central to OMV production, and potentially linked to vesiculation mechanisms (20). The one protein identified that was enriched in the SL-null OMVs was identified as Arg-gingipain (PG0506, RgpB; Table 1). Although this was not a comprehensive analysis of proteins carried on SL-plus and SL-minus OMVs, such differences in the protein content support our hypothesis that like lipid rafts in eukaryotes (21), SL-containing microdomains in the outer membrane of P. gingivalis contain select proteins, which are ultimately packaged in SL-OMVs, thereby contributing to the ability of SL-OMVs to be immunosuppressive.

**TABLE 1:** Spots selected for further analysis from the comparative fluorescent two-dimensional gel of OMV protein cargo from WT P. gingivalis W83 (green) and the W83 SPT mutant (ΔPG1780; red)

Spot no.	P. gingivalis strain W83 ID^*a*^	Annotation	Strain with higher abundance in OMVs^*b*^	
			WT W83	W83 ΔPG1780
5	PG1881	Pilin-forming lipoprotein	X	
6	PG1881	Pilin-forming lipoprotein	X	
7	PG1881	Pilin-forming lipoprotein	X	
8	PG1881	Pilin-forming lipoprotein	X	
50	PG0506	Arginine-specific cysteine protease (RgpB)		X
60	PG1881	Pilin-forming lipoprotein	X	
65	PG1881	Pilin-forming lipoprotein	X	

a ID, identifier.

b The strain with a higher abundance of annotated protein in its OMVs is indicated by an X.

### Immunomodulatory effect of P. gingivalis SL synthesis occurs without interference of active gingipains.

Gingipain activity is known to modulate the ability of the host to mount a proper immune response by degrading immunologically important molecules (22); therefore, we assessed the possibility of interference of active gingipains in our assay. Gingipain assays (both arginine and lysine specific) performed on THP-1 cell culture supernatant fluids showed only nominal gingipain activity without addition of a reductant (normoxic conditions), and similar levels of activity were detected in the cell culture supernatant fluids of WT and SPT- mutant when tested under typical reducing conditions (10 mM cysteine) as a control (see Fig. S1 in the supplemental material). Inspection of gingipain activity from bacterial culture supernatant fluids and cell pellets from WT and SPT- P. gingivalis revealed that the culture supernatants of P. gingivalis SPT- mutant possessed slightly higher, not lower levels of gingipain activity compared with WT (Fig. S1), while similar levels of gingipain activity were observed between WT and SPT- mutant cell pellets (Fig. S1). Our findings show that the observed low-level inflammation observed in response to WT P. gingivalis in the transwell experiments does not align with elevated gingipain activity.

### Purified OMVs suppress the host cell inflammatory response.

**FIG 3:**
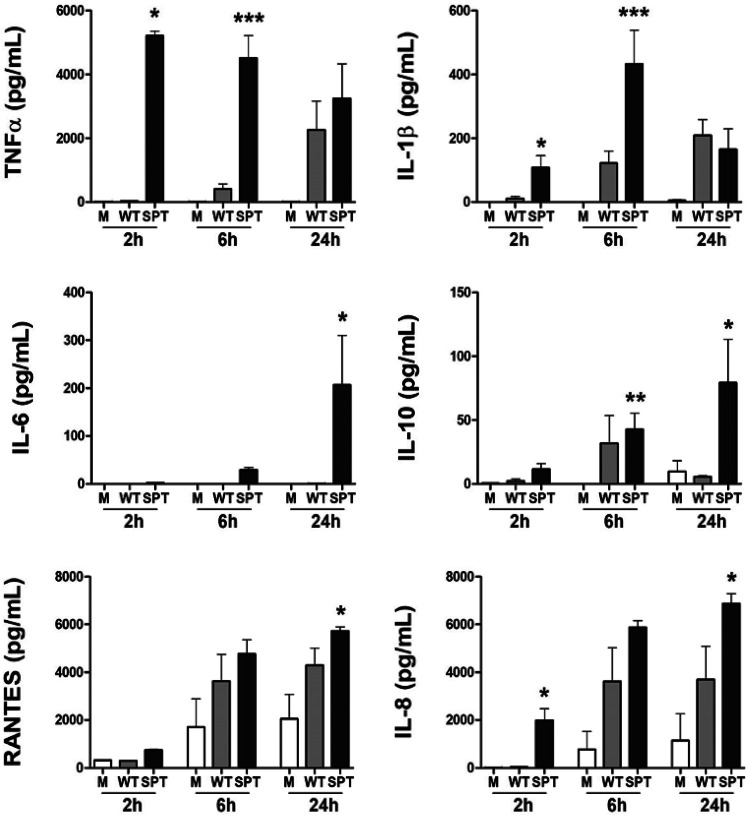
Purified OMVs from P. gingivalis from SL-null mutant elicited higher cytokine and chemokine response. PMA-treated human macrophage-like THP-1 cells were directly cultured with purified OMVs (1,000 particles/cell) from P. gingivalis W83 (WT; gray bars) or the P. gingivalis W83 SL-null mutant (SPT-; black bars). Supernatant fluids were collected at 2, 6, and 24 h, and the levels of TNF-α, IL-1β, IL-6, IL-10, RANTES, and IL-8 were measured by multiplex immunoassay. Medium alone (M; white bars) served as unchallenged control. Data are presented as means plus SEM (*n* = 4 independent experiments). Values that are significantly different from the value for WT P. gingivalis using unpaired *t* test are indicated by asterisks as follows: *, *P* < 0.05; **, *P* < 0.01; ***, *P* < 0.005.

To evaluate the possibility that purified P. gingivalis OMVs are able to recapitulate the immune stimulating activity previously observed by direct bacterial challenge (17), and no-contact/transwell challenge, purified OMVs from WT and SPT- mutant were added directly to THP-1 cells. We detected a hyperinflammatory response from THP-1 cells cultured directly with OMVs from the SL-null mutant in comparison to the elicited response by the OMVs from the WT (Fig. 3). The levels of tumor necrosis factor alpha (TNF-α) and interleukin 1β (IL-1β) were significantly higher at 2 h and 6 h after stimulation with the SL-null mutant than after stimulation with the WT (*P* < 0.05 for all by *t* test), and that same trend was evident at 24 h. The OMVs produced by the SL-null strain also elicited higher levels of IL-6, IL-8, RANTES, and IL-10, with a significant increase observed at 24 h (Fig. 3). These findings confirm that purified P. gingivalis OMVs recapitulate the findings for suppression of the immune response to P. gingivalis observed when we evaluated systems using whole live bacteria.

## DISCUSSION

The concept that P. gingivalis SLs participate in modulating host inflammation during infection is a critical point of novel understanding that is further supported and extended in the present study. As P. gingivalis SLs have been recently shown to be transferred to host cells, and this transfer correlates with suppression of the inflammatory response from those cells (17), we speculated that SL-OMVs act as a delivery platform for transfer. It is understood that P. gingivalis is a robust producer of OMVs, that these nanostructures can elicit inflammatory responses (3), and that the mechanism of elicited inflammation is in part dependent on the innate immune sensing of these particles by host cells (6, 19). However, there is still a gap in the knowledge of the role of OMVs from the different type strains of P. gingivalis and in inflammatory immune cells, such as macrophages. Cecil et al. (6) found that OMVs from P. gingivalis strain W50 strongly interacted with monocytes and macrophages, increasing their proinflammatory responses, in a dose-dependent manner. Lower concentrations of OMVs induced stronger immune response, while higher concentrations were less inflammatory in unprimed cells, but stronger upon second exposure. OMVs from P. gingivalis strain W50 have also been shown to induce a metabolic shift in macrophages, with strong activation of proinflammatory cytokine production, inflammasome signaling, and pyroptotic cell death, in contrast to a weak cytokine production, with no inflammasome activation or pyroptosis induction by macrophages that were stimulated with the whole bacteria (23). In this study, we demonstrated that macrophages cultured with purified OMVs from P. gingivalis strain W83 produced a mild inflammatory phenotype consistent with live bacterial challenge, while OMVs from the SPT- mutant were found consistent with a hyperinflammatory phenotype when the SLs were absent. More studies are required to obtain a better understanding of how different strains of P. gingivalis impact the interaction between OMV and host cells.

The fact that OMVs deliver P. gingivalis SLs to the host cell is particularly fascinating when we consider the periodontal environment, since previous studies uncovered that microbial SLs are present at differing levels in gingival tissues of healthy individuals and patients with periodontal diseases (24). Intriguingly, bacterial SLs seem to go through a shift in their dihydroceramide (DHC) pools that matches the transition from health to disease. In the diseased gingival tissues, the lipid pools present low phosphoglycerol-DHC (PG-DHC) lipid levels and an increase in phosphoethanolamine-DHC (PE-DHC) lipids (24). These findings suggest that synthesis of different SLs by the oral *Bacteroidetes* may influence periodontal homeostasis as well as disease progression; thus, the different SL pools may be related to both conditions of the gingival tissues. *In vitro* studies using isolated P. gingivalis SLs have shown effects of purified individual SLs in bone remodeling and inflammatory expression in some cells (18, 25–30), and our recently published data, utilizing the whole bacteria. These findings support the fundamental role of P. gingivalis SLs in immune regulation (17), suggesting that SLs have multiple functions. Future research, including studies aimed at defining SL impacts on other immunologically important cell types, including neutrophils and others, as well as animal modeling is needed to decipher the importance of specific P. gingivalis SLs in the context of infection-elicited host response and linkage to periodontal disease.

In an earlier study, we determined that WT P. gingivalis OMVs contain SLs and that the STP- mutant was devoid of SLs (31). Here, we explored the impact of SL synthesis on the characteristics of OMVs produced by P. gingivalis by comparing OMVs from the SL-producing WT and SL-null mutant. The OMV size distribution that we observed is comparable to that detected by others, which was reportedly to range from 50 to 300 nm in diameter (19, 32). We observed that P. gingivalis produced OMVs even when it cannot synthesize SLs and that OMV production was greater in the SL mutant than WT, suggesting that SL synthesis influences biogenesis of OMVs that do not contain SLs or that SL synthesis negatively regulates production of other OMVs that do not possess SLs. Like other bacterial OMVs, the OMVs of P. gingivalis are known to possess an array of molecules, including proteins and LPS, as well as its peptidoglycan (33). To begin to understand the contribution of SLs to the loading of cargo to purified OMVs, we compared the protein profiles between wild-type and SL-null OMVs and noted some dramatic differences, indicating that SL synthesis impacts OMV cargo. Recent publications have shown that proteins carried on OMVs produced by P. gingivalis strain W83 can be citrullinated by peptidylarginine deiminase (PPAD) and this included protein PG1881 (20). The data presented in this study demonstrate that when P. gingivalis cannot synthesize SLs, it was proficient in OMV biogenesis, yet the PG1881 protein is absent (or at low abundance) in the OMVs. Since, as noted, we previously determined that P. gingivalis OMVs can contain SLs (31), this finding suggests that SL-containing OMVs are enriched in PG1881. Importantly, a recent report (34) showed that PG1881 is a predicted pilus-forming lipoprotein that plays a role in vesiculation. Thus, our working model is that certain subtypes of SLs form membrane microdomains which direct localization of PG1881 to these SL-rich areas. Studies are under way to elucidate the mechanisms involved in SL-OMV biogenesis.

**FIG 4:**
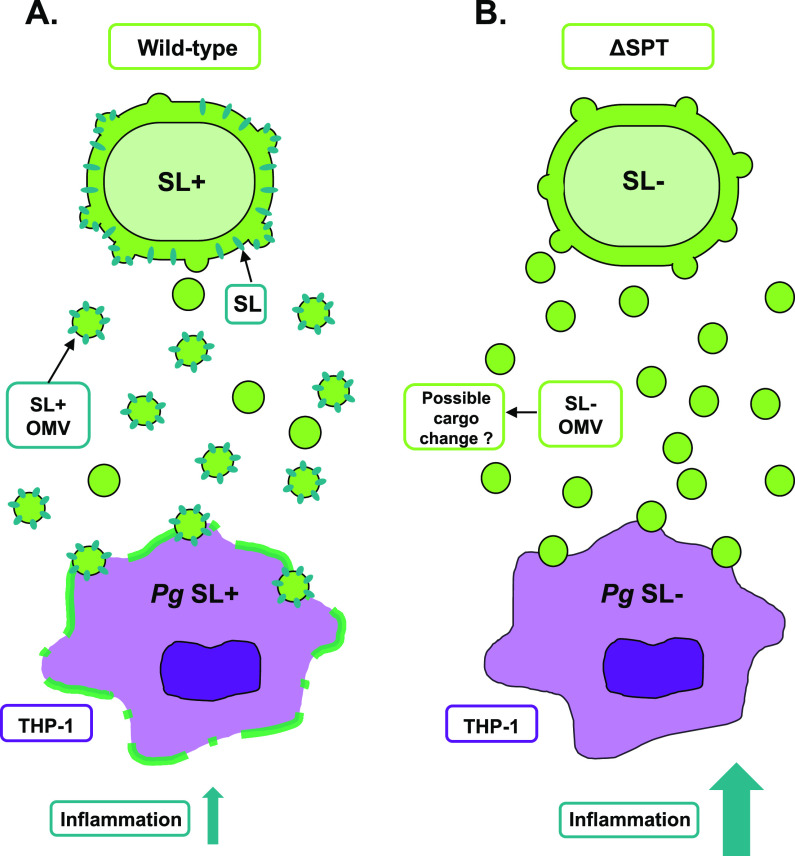
Proposed working model elucidating the role of P. gingivalis OMVs as a vehicle to deliver bacterial sphingolipids (SLs) to host cells. In a transwell system, wild-type P. gingivalis (*Pg*) is able to deliver its SLs to THP-1 cells in a contact-independent manner (A). The SL-null mutant (ΔSPT) still produces OMVs (B), but as SLs are not synthesized, it elicits a hyperinflammatory response by the host cells in comparison to the wild type, demonstrating the participation of P. gingivalis SLs or a change in OMV cargo as a consequence of changed SL levels in the regulation of inflammatory response by the host cells.

As previously demonstrated, the direct challenge with the SL-null mutant elicited a hyperinflammatory profile from the host cells (17). Here, a highly similar cytokine profile was detected from macrophages cultured with wild-type and SL-deficient P. gingivalis when bacterium-to-host cell contact was prevented by using a transwell system (Fig. 4). The inflammatory profile detected in this study using wild-type P. gingivalis has a close similarity to that found in periodontal disease, with elevated levels of proinflammatory cytokines TNF-α, IL-1β, and IL-6 and chemokines such as IL-8, which are characteristically found in gingival tissues of periodontal disease patients (35). We have also found higher levels of IL-10, an anti-inflammatory cytokine that has been shown to be upregulated in gingival crevicular fluid of periodontal patients and known to suppress the production of proinflammatory cytokines from several cell types (36, 37). The inflammatory response was exacerbated by the absence of SLs in a contact-independent manner, suggesting that OMVs containing SLs are capable of regulating inflammation, and this ability was impacted by the different cargo loading of the SL-null OMVs, consequently eliciting a hyperinflammatory profile. The mechanisms by which this occurs are beginning to be explored. Interestingly, our findings are consistent with other closely related bacteria such as Bacteroides thetaiotaomicron in the intestinal tract, where B. thetaiotaomicron SL production and uptake by host tissues is important to maintain gut homeostasis as a SL-deficient mutant elicited significantly larger amounts of inflammation in an inflammatory bowel diseases model (30, 38). Although it is not clear how P. gingivalis OMVs deliver their cargo to the host, we demonstrate here that purified OMVs from SL-null mutant elicit a stronger inflammatory response than OMVs from wild-type P. gingivalis. These findings produce the same inflammatory profile we detected when using the whole bacteria, by both direct challenge and in the contact-prevented transwell system. Interestingly, when stimulated with purified OMVs, the stronger inflammatory response by THP-1 cells is evidently higher, compared to the whole molecule infection, and can also be detected in our earliest evaluated time point, at 2 h. It is noteworthy that SL-containing OMVs can limit the inflammatory response of the host like the whole bacteria, indicating the fundamental role of P. gingivalis OMV cargo in its interaction with the host cells. Strong interaction of OMVs from P. gingivalis with THP-1 macrophages has been reported, including induction of phagocytosis, NF-κB activation, cellular priming, and accentuated proinflammatory response (6). It is also well understood that OMVs can penetrate and disseminate through host tissues more easily than their larger parent bacterial cells, due to their nanoparticle size and proteolytic and adhesive properties (39).

Our past work, and current studies have shown that TLRs (TLR2, TLR4, and others) and their signaling pathways are important in host innate immune sensing of P. gingivalis (40–43). Comparing the THP-1 cell response to SL-containing and SL-deficient P. gingivalis in the transwell system, we found that THP-1 cells cultured with SL-containing P. gingivalis presented reduced expression of TLR2 gene and TLR adaptor molecules MyD88 and TICAM1/TRIF compared with the hyperinflammatory SL-deficient mutant; however, no differences in TLR4 gene expression were observed. Both TLR2 and TLR4 are critical receptors to control the induction of TNF-α, IL-1β, IL-6, and IL-8 by pathogens in the periodontal tissues (44), to P. gingivalis by cells exposed to the organism *in vitro* (41, 42, 45, 46), and it is also known that P. gingivalis OMVs utilizes TLRs *in vitro* (19). It is well established that TLR2 and TLR4 and their signaling pathways (MyD88 and TRIF) play an important role in how P. gingivalis is immunologically sensed by the host, and purified SLs have also been reported to signal through TLR2 (47, 48). In addition, it has been suggested in a study using purified P. gingivalis OMVs that these particles may be critical to inflammasome activation and stimulation of multiple pattern recognition receptors (PPRs), which may have a synergistic effect, perhaps even greater than the stimulation of TLR4 alone (19). Thus, further studies are necessary to obtain a better understanding of the mechanisms and specific pathways that control the immunosuppressive effect we observe in the presence of P. gingivalis SLs.

Although several pathways exist by which P. gingivalis can promote immune dysregulation (49), a well-established mechanism utilized by P. gingivalis is the degradation of many host proteins by gingipains, which are cysteine proteinases (50). Considered major virulence determinants of P. gingivalis, the arginine and lysine gingipains can be released from the organism via OMVs or directly secreted. Previous work from our group identified that the SL-null mutant produces lower cell-associated arginine and lysine gingipain activity compared to the WT, with an increase in the release of gingipains into the supernatant (31). As we noted that there are protein cargo differences of OMVs from WT and SL- P. gingivalis, we also examined gingipain activity, both under conditions resembling the cell culture environment (normoxia) and using classical highly reducing conditions. Interestingly, almost no gingipain activity was detected in the cell culture supernatants when THP-1 was cultured with the wild type or with SL-null mutant in a transwell system. These findings showed that under cell culture conditions gingipain activity was essentially nonexistent; however, when these same samples were examined under a reducing environment of traditional assay conditions, low levels of gingipain activity could be detected. Importantly, there was no difference in activity between WT and SL-mutant transwell supernatant fluids. In parallel, bacterial cell culture supernatant fluids and cell pellets grown in the same medium (RPMI 1640) were used to validate the assay. The SPT mutant supernatant possessed higher levels of gingipain activity compared with WT P. gingivalis, as previously shown when the strains were grown in Trypticase soy broth (TSBHK) (31). The importance of these data are that the supernatant fluids, where OMVs would be enriched, do not have more activity in the SPT mutant than in the WT. Although this does not eliminate a role for gingipains, the findings indicate that a reduction in gingipain activity is not a likely explanation for the observed elevated levels of inflammatory molecules in response to P. gingivalis when the organism is unable to synthesize SLs. Our data, therefore, suggest that the mechanism of immunomodulation is mediated directly by the SLs on the OMVs or the cargo of the OMVs or both; however, the underlying mechanism(s) remains to be determined.

In the context of periodontal disease, macrophages participate with 5 to 30% of the local inflammatory infiltrate, and its differentiation is known to be crucial to start the process of bone resorption *in vivo* (51). Further, in animal modeling, depletion of macrophages (52), or macrophage reconstitution assays (42) have confirmed a central role for macrophages in P. gingivalis-elicited oral bone loss. In an infected periodontium, OMVs can disrupt epithelial cell junctions and deliver virulence factors from bacteria to immune cells that are in the underlying tissues. Therefore, the concentration gradient of OMVs is likely to occur from the site of bacterial infection in the polymicrobial biofilm adhered to the tooth root. OMV concentrations then will tend to be lower at distal areas from the initial site of infection, priming the host cells, but not necessarily promoting cell activation. As the disease progresses, the tissues previously primed by exposure to OMVs will consequently have a more robust inflammatory response (6). Considering that these proinflammatory interactions depend upon early OMV interactions with macrophages and possibly other immune cells, our data reveal that P. gingivalis SLs delivered by OMVs have an important role in the regulation of that process. We suggest that P. gingivalis OMVs initiate pathogen recognition and/or inflammatory signaling in a way that can be limited by its synthesis of certain subtypes of SLs. While the molecular mechanisms utilized by P. gingivalis to exist in periodontal health and in disease remain incompletely defined, this duality requires further investigations to better understand how this organism utilizes SL-containing OMVs to manipulate the host-pathogen interaction.

## MATERIALS AND METHODS

### Bacterial growth and conditions.

For these studies we used wild-type (WT) P. gingivalis strain W83, and a previously characterized isogenic mutant incapable of producing SLs due to deletion of the PG1780 gene encoding the enzyme serine palmitoyl transferase (W83 ΔPG1780; SPT-) (31). Bacteria were cultivated on blood agar plates (BAPHK), and in Trypticase soy broth (TSBHK) at 37°C in an anaerobe chamber. Bacteria were harvested from broth culture by centrifugation, washed three times with RPMI 1640 medium adjusted to approximately 4 × 10^9^ CFU/ml and were added to the upper well of the transwell cell culture system to achieve a ratio of 2,000 bacteria per host cell.

### Cell culture.

The human monocyte cell line THP-1 (TIB-202; ATCC, Manassas, VA) was cultured in RPMI 1640 (Corning) supplemented with 10% heat-inactivated fetal bovine serum at 37°C in a 5% CO_2_ incubator as we have done previously (17). THP-1 cells were adjusted to 5 × 10^5^ viable cells/ml and placed into fresh medium with 100 ng/ml phorbol 12-myristate 13-acetate (PMA; Sigma-Aldrich, St. Louis, MO) to induce differentiation into a macrophage-like state. After 48-h incubation, and cell washing, sterile 0.4-μm transwell inserts were placed in the wells with THP-1 cells and the inserts were filled with 125 μl of medium, P. gingivalis W83 parent strain or SPT- mutant as described above. After 2, 6, and 24 h, the cell culture supernatant fluids were collected and the levels of TNF-α, IL-1β, IL-6, IL-8, IL-10, and RANTES were determined by Milliplex Multiplex Assays (EMD, Millipore, Billerica, MA). Data were acquired on a Luminex 200 system running xPONENT 3.1 software (Luminex, Austin, TX) and analyzed using a five-parameter logistic spline curve-fitting method using Milliplex Analyst V5.1 software (Vigene Tech, Carlisle, MA).

### P. gingivalis OMV purification and characterization.

WT P. gingivalis W83 and the SPT- mutant were cultured in broth media as described above, and OMVs were isolated and characterized as previously described (19) with modifications. Bacteria were grown initially in TSBHK medium before being grown in RPMI 1640 medium under anaerobic conditions at a final volume of 500 ml. The cultures were moved to an aerobic incubator for 6 h prior to harvesting to mimic conditions of the transwell experiments. Cultures were clarified by centrifugation at 7,000 × *g* for 30 min. The supernatant was filter sterilized (0.2-μm-pore-size PES membrane), and then clarified supernatants were concentrated by ultrafiltration at 40°C using a Millipore stirred ultrafiltration apparatus (Millipore-Sigma, Burlington, MA). Concentrated supernatants were then ultracentrifuged at 100,000 × *g* for 2 h at 40°C to pellet crude OMVs, and the pellets were resuspended in 45% OptiPrep (Sigma-Aldrich) density gradient medium in HEPES buffer and overlaid with a continuous OptiPrep density gradient (45% to 15%) and centrifuged at 100,000 × *g* for 16 h. The fraction(s) containing the OMVs was suspended in HEPES buffer and then centrifuged at 100,000 × *g* for 2 h. The resultant purified OMV pellet was resuspended in a minimal volume of HEPES. Particle enumeration and size distribution were determined by NanoSight NS300 (Malvern Panalytical, Malvern, UK). The concentration of OMVs was normalized, and the OMVs were added directly to THP-1 cells at a ratio of 1,000 particles per host cell, as previously described (53).

### Two-dimensional difference gel electrophoresis analysis of OMVs.

OMVs were isolated and purified and submitted to Applied Biomics, Hayward, CA, for two-dimensional difference gel electrophoresis (2D-DIGE) protein analysis. OMV counts were determined by NanoSight quantification, and each sample was normalized to contain equal amounts of vesicles prior to submission as centrifuged pellets. Proteins in OMV samples were labeled with the following fluorescent dyes: Cy2 (red) parent strain W83 and Cy3 (green) for the corresponding W83Δ1780 SL-null mutant strain. The spectrally resolvable dyes were simultaneously separated on a single 2D gel, with isoelectric focusing (IEF) in the first dimension and sodium dodecyl sulfate (SDS)-polyacrylamide gel electrophoresis in the second dimension. After electrophoresis, the gel was scanned using a Typhoon image scanner. Each scan reveals one of the CyDye signals (Cy2 or Cy3). ImageQuant software was then used to generate the image presentation data, including the single and overlay image (Fig. 2C). Comparative analysis of all spots was done using DeCyder “in-gel” or “cross-gel” analysis software. The OMV protein content ratios between different samples were generated. Spots of interest (distinct ratios as determined by the DeCyder software) were automatically picked from the 2D gel with the Ettan Spot Picker and stored. Seven protein spots (spots 5, 6, 7, 8, 50, 60, and 65) that demonstrated high differential abundance between the parent strain W83 and W83Δ1780 OMVs were identified by Applied Biomics using mass spectrometry.

### Gingipain activity assays.

The activity of arginine and lysine gingipains was assessed as previously described (31, 54). Cultures were inoculated from a plate into TSBHK, grown for 24 hand then diluted into RPMI 1640 for experimental samples. Cultures were grown to exponential phase, normalized to an optical density at 600 nm (OD_600_) of 1.0, and 1 ml of each culture was centrifuged. The supernatant fluids of these cultures were saved and assayed, and the pellets were resuspended in assay buffer (200 mM Tris, 5 mM CaCl_2_, 150 mM NaCl, either in the presence or absence of 10 mM l-cysteine at pH 7.6). Cultures and supernatants were diluted 1/10 in assay buffer and then serially diluted in assay buffer across a 96-well microtiter plate. The initial optical density (OD_405_) was recorded, and the plates were placed in a 37°C incubator for 10 min to equilibrate the temperature. *N*-α-Benzoyl-l-arginine-*p*-nitroanilide (BAPNA) or *N*-α-acetyl-l-lysine-*p*-nitroanilide (ALPNA) was added to the wells at a final concentration of 1 mM, and the microtiter plates were incubated for 2 h at 37°C. The final optical density of the wells was recorded, and the difference between the initial and final optical density was reported.

### Quantitative real-time PCR.

Total RNA was extracted from cell lysates using RNeasy kit (Qiagen, Germantown, MD) according to the manufacturer’s protocols. The quantity and purity of RNA was determined by UV spectrophotometry using the 260/280 nm ratios. For each sample, 500 ng of total RNA was converted into cDNA using a High Capacity cDNA Synthesis kit (Applied Biosystems, Foster City, CA). The quantitative PCRs (qPCRs) were performed in a 20-μl total volume reaction, utilizing TaqMan qPCR master mix (TaqMan Fast Advanced, ThermoFisher, Waltham, MA), cDNA template, deionized water, and human-specific predesigned primers and probe for TLR2, TLR4, MyD88, TICAM/TRIF, and the housekeeping gene β-actin (TaqMan gene expression assays, ThermoFisher). Cycling conditions were preoptimized by the supplier, and 40 cycles were run on a StepOne Plus qPCR thermocycler (Applied Biosystems). Relative levels of gene expression were determined by the ΔΔ*C_T_* method using the thermocycler’s software and automated detection of the cycle threshold (*C_T_*). Expression levels of β-actin in the same samples were used to normalize results.

### Statistical analysis.

Data were obtained from at least three independent experiments. Immunoassay and gene expression data were collected, and both descriptive and comparative statistical analyses were performed using GraphPad 8.0 (GraphPad; San Diego, CA), nonpaired *t* test with Welch’s correction for unequal variances, and the significance level was set at 95% (*P* < 0.05) for all analyses.

## Supplementary Material

Supplemental file 1
