# Supplementary material for: Sphingolipid-Containing Outer Membrane Vesicles Serve as a Delivery Vehicle To Limit Macrophage Immune Response to Porphyromonas gingivalis
Source: Infect Immun. 2021 Mar 17;89(4):e00614-20. doi: 10.1128/IAI.00614-20 (PMC8090959; doi:10.1128/IAI.00614-20)
Supplement: Supplemental file 1 [file IAI.00614-20-s0001.pdf]

Supplemental Figure 1

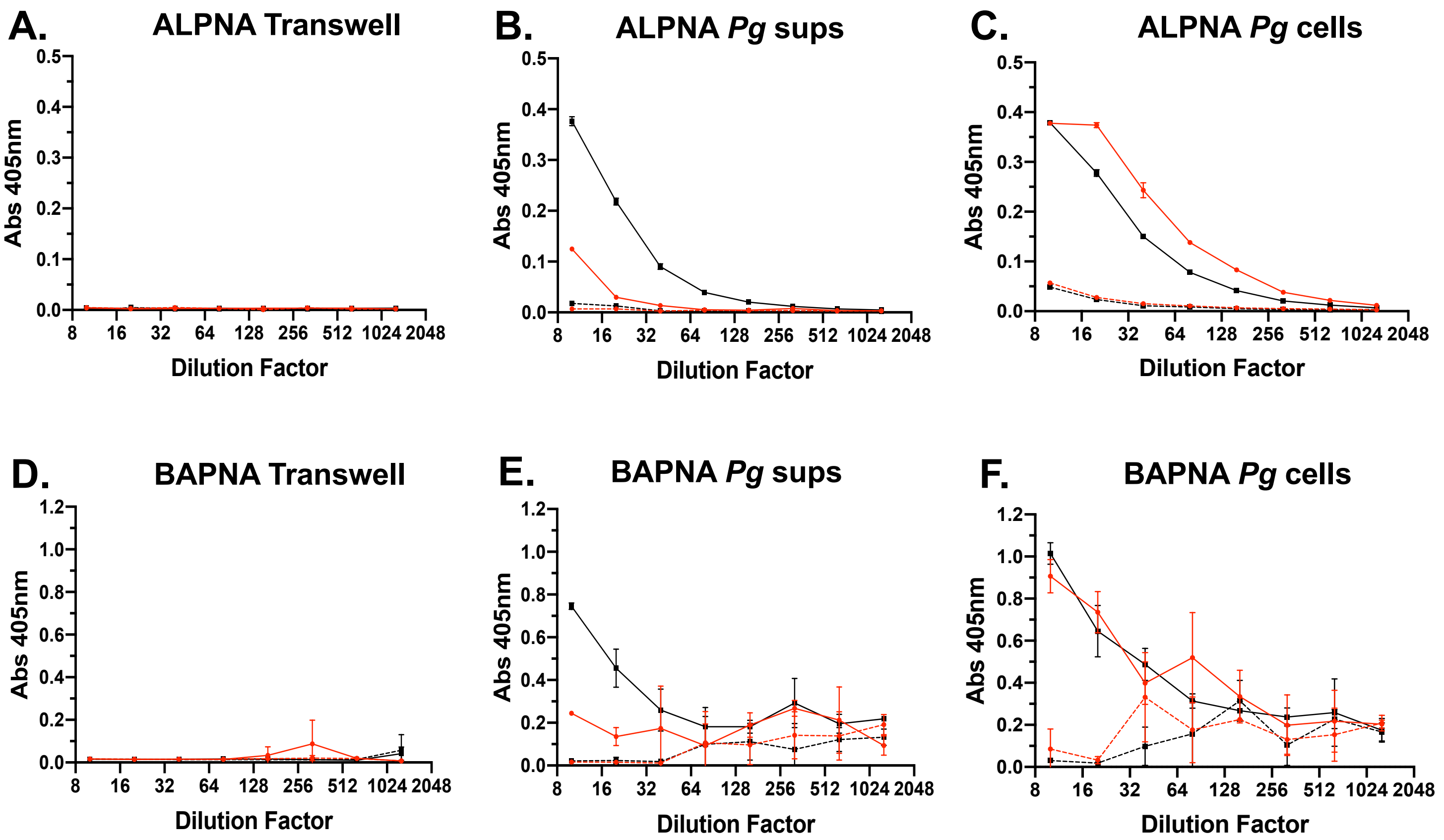

Supplemental Figure 1. Gingipain activity present in transwell system, as we all *P. gingivalis* culture supernatant fluid and cell pellet. Culture supernatant fluids were collected from the bottom chamber of transwell experiments (**A.** and **D.**), as well as from both the bacterial cell culture supernatant fluids after separation from bacterial cells by centrifugation (**B.** and **E.**) and from the accompanying *P. gingivalis* cell pellets (**C.** and **F.**) these samples were assayed for lysine gingipain (ALPNA; **A.**, **B., and C.**) and arginine gingipain (BAPNA; **D., E., and F.**) activity. Transwell culture supernatant fluid levels for gingipain activity were low for W83 (red traces) and SPT- mutant (black traces) whether exogenous L-cysteine was absent (dashed lines) or present (solid lines) in the assay medium. Culture fluids collected from *P. gingivalis* following growth in TSBK, and incubation in RPMI-1640 under cell culture conditions in the absence of host cells for 6 hrs. were found to have low gingipain activity in the absence of supplemental L-cysteine addition; however, it was noted in both arginine- and lysine-gingipain activity was highest in culture supernatants of SPT- mutant compared with WT *P. gingivalis*. Cell pellets of *P. gingivalis* corresponding to these bacterial supernates following 6 hrs. incubation in RPMI-1640 were found to have robust arg and lys gingipain activity with W83 (red trace) possessing elevated lys-gingipain activity compared with SPT- mutant (black trace) yet possessed similar arg-gingipain activity to SPT- when assayed in supplemental L-cysteine (solid lines). Low gingipain activity was detected in the absence of supplemental L-cysteine addition (dashed lines). Data presented as mean +/- SD for n=3 separate measurements.
